# Early and late skin reactions to radiotherapy for breast cancer and their correlation with radiation-induced DNA damage in lymphocytes

**DOI:** 10.1186/bcr1277

**Published:** 2005-07-01

**Authors:** Escarlata López, Rosario Guerrero, Maria Isabel Núñez, Rosario del Moral, Mercedes Villalobos, Joaquina Martínez-Galán, Maria Teresa Valenzuela, José Antonio Muñoz-Gámez, Francisco Javier Oliver, David Martín-Oliva, José Mariano Ruiz de Almodóvar

**Affiliations:** 1 Servicio de Oncología Radioterápica, Hospital Universitario Virgen de las Nieves, Granada, Spain; 2 Instituto de Biopatología y Medicina Regenerativa, Centro de Investigaciones Biomédicas, Departamento de Radiología y Medicina Física, Facultad de Medicina, Universidad de Granada, Granada, Spain; 3 Instituto de Parasitología y Biomedicina 'López Neyra' CSIC, Parque Tecnológico de Ciencias de las Salud, Granada, Spain

## Abstract

**Introduction:**

Radiotherapy outcomes might be further improved by a greater understanding of the individual variations in normal tissue reactions that determine tolerance. Most published studies on radiation toxicity have been performed retrospectively. Our prospective study was launched in 1996 to measure the *in vitro *radiosensitivity of peripheral blood lymphocytes before treatment with radical radiotherapy in patients with breast cancer, and to assess the early and the late radiation skin side effects in the same group of patients. We prospectively recruited consecutive breast cancer patients receiving radiation therapy after breast surgery. To evaluate whether early and late side effects of radiotherapy can be predicted by the assay, a study was conducted of the association between the results of *in vitro *radiosensitivity tests and acute and late adverse radiation effects.

**Methods:**

Intrinsic molecular radiosensitivity was measured by using an initial radiation-induced DNA damage assay on lymphocytes obtained from breast cancer patients before radiotherapy. Acute reactions were assessed in 108 of these patients on the last treatment day. Late morbidity was assessed after 7 years of follow-up in some of these patients. The Radiation Therapy Oncology Group (RTOG) morbidity score system was used for both assessments.

**Results:**

Radiosensitivity values obtained using the *in vitro *test showed no relation with the acute or late adverse skin reactions observed. There was no evidence of a relation between acute and late normal tissue reactions assessed in the same patients. A positive relation was found between the treatment volume and both early and late side effects.

**Conclusion:**

After radiation treatment, a number of cells containing major changes can have a long survival and disappear very slowly, becoming a chronic focus of immunological system stimulation. This stimulation can produce, in a stochastic manner, late radiation-related adverse effects of varying severity. Further research is warranted to identify the major determinants of normal tissue radiation response to make it possible to individualize treatments and improve the outcome of radiotherapy in cancer patients.

## Introduction

Ionizing radiation is widely and successfully applied in oncology. However, because of dose restrictions, a definitive cure cannot be achieved for many tumour entities and localizations. Despite the advanced radiotherapy facilities available, high doses of radiation still induce early and late skin effects. Unacceptable normal tissue reactions remain the limiting factor for delivering a tumoricidal dose in radiotherapy. Radiation is an unusual toxic agent in that the timing of tissue damage expression can vary widely between one tissue or tumour and another [1]. On the other hand, recent large-scale trials of adjuvant radiotherapy for breast cancer showed that the overall survival benefit of radiotherapy can be considered an inherent characteristic of the treatment and is not influenced by the duration of follow-up [2]. Data in the literature strongly support a causal relation between better outcomes and improved radiotherapeutic techniques [3]. Changes in radiotherapy practice over the years include recognition of the importance of fraction size, fraction number, total dose, overall time for both tumour and normal tissue reactions, and the introduction of conservative therapy.

Radiotherapy outcomes might be further improved by a greater understanding of the individual variations in normal tissue reactions that determine tolerance [4]. When accurate genetic-based or cell-survival-based predictive assays are available to study tumour and normal tissue radiosensitivity, radiation therapy will become an exact science [5], allowing truly individual optimization and the prediction of adverse reactions [6]. It is of great importance to identify the variations in intrinsic (cellular) radiosensitivity and extrinsic factors that are associated with a change in the risk of morbidity. It has yet to be determined whether intrinsic cell radiosensitivity or extrinsic factors have greater influence on individual differences in damage expression [7-10]. The very high incidence of breast cancer in Western countries, partially attributable to the ageing of their populations, and the increasing use of conservative surgery and postoperative radiotherapy for its treatment make the above type of study of special interest, with the side effects of radiotherapy an increasingly important issue. Indeed, after the sweeping changes in the locoregional treatment of breast cancer during the last part of the 20th century, it appears that only a dwindling minority of patients will undergo mastectomy, at least in urban areas with a high socioeconomic level [11]. The widely varied biological characteristics of patients with breast cancer, evidenced in clinical, pathological, cellular, and molecular studies, are sufficient to explain the diversity of treatments recommended over the past two decades [12]. Recent years have seen the introduction of changes from conventional radiotherapy at 5 × 1.8 to 2.0 Gy per week to more aggressive schedules such as unconventional protocols [13] or radiochemotherapy [11]. The gradually increasing success of cancer treatments has led to longer patient survival. This also carries with it the penalty of providing a greater opportunity for late effects to appear, increasing in severity [14] and affecting the patient's quality of life [15].

With regard to radiotherapy complications, the known factors influencing normal tissue responses account for only 30% of interpatient variability in breast cancer patients under well-controlled conditions, leading to the hypothesis that most of the variability in the severity of these complications is due to differences in cellular radiosensitivity determined by genetic or epigenetic mechanisms [7,10]. Identification of the causes of this variability in radiation sensitivity could have important implications for cancer therapy. Evidence of a possible genetic basis for these differences has been provided by reports of increased cellular and tissue radiosensitivity in certain genetic syndromes [16] and of an association among the relative radiosensitivities of different normal cell types in the same individual [17]; this evidence also verifies that cellular radiosensitivity may be related to tissue response. Current radiobiological research efforts are aimed at identifying patients with abnormal radiosensitivity at risk for acute and late adverse effects of radiotherapy treatment [18,19] and detecting molecules that increase the antitumour effects of radiotherapy [20].

Most published studies on radiation toxicity were performed retrospectively. This prospective study was launched in 1996 to measure the *in vitro *radiosensitivity of peripheral blood lymphocytes before treatment with radical radiotherapy in patients with breast cancer, and to assess the early and the late side effects of radiation on skin in the same group of patients. We prospectively recruited consecutive breast cancer patients receiving radiation therapy after breast surgery. To evaluate whether early and late side effects of radiotherapy can be predicted by the assay, a study was conducted of the association between the results of *in vitro *radiosensitivity tests and acute and late adverse effects of radiation.

## Materials and methods

### Patients

The data analysed in this study were derived from 108 consecutive breast cancer patients who received radiotherapy and were followed up for 7 years within our departmental program for the predictive testing of the radiosensitivity of normal tissue. The investigation was approved by the local ethics committee, and written, informed consent was obtained from all patients. Patient recruitment started in March 1996. Late adverse skin effects were measured between December 2003 and June 2004. The study design and patient and treatment characteristics have been published previously [9].

The patients were treated with postoperative radiation therapy after mastectomy (54 patients) or with breast-conserving therapy using a standardized ^60^Co technique (54 patients). The dose delivered was 50 Gy over a period of 5 weeks, in daily fractions of 2 Gy (25 fractions at 5 per week). External radiation was delivered by the cobalt unit in almost all of the patients (98%), and only 2% received irradiation from a Linac 6-MV x-ray linear accelerator. The whole breast or chest wall was irradiated by two parallel, opposed tangential fields, with wedges used to correct dose inhomogeneities. The dose was prescribed at the ICRU (International Commission on Radiation Units and Measurements) point at the midline of the central axis. Dose homogeneity was more than 85% in the majority of the cases. Patient treatments were planned using computed tomography images and a conventional simulator. To administer regional nodal radiation, we used a direct anterior field to irradiate internal mammary nodes. Supraclavicular and axillary lymph node areas were treated by irradiation of the axillary–supraclavicular field and the posterior axillary field. The total dose was calculated at 3 cm in the supraclavicular area and at the midplane in the axilla. The conservatively treated patients also received a tumour bed boost of 16 to 25 Gy using an iridium implant (^192^Ir), always 15 days after external radiotherapy or electron beam therapy. The ^192^Ir implants were done in accordance with the rules of the Paris System of Dosimetry. The dose was calculated at the reference isodose, defined as 85% of the basal dose calculated in the central plane of application. The total dose delivered by 9-to 12-MeV electron beams was 16 Gy at 2 Gy per fraction. The dose was prescribed to the 90% isodose line. A bolus was sometimes used to optimize the homogeneity of dose distribution.

All medical records of these 108 patients were available and were reviewed. Patient files included details of surgery, clinical-pathological stage, adjuvant treatment, and the subsequent follow-up. The records also included full details of the radiotherapy treatment, and a photograph of the irradiated field was always made on the last treatment day to record the intensity of the acute radiation-induced injury on the skin of each patient.

### Definitions of descriptive terms for skin reactions

The severity of skin reactions was assessed by means of a simple scale (Table 1), using scores based on the absolute side-effect scale proposed by the Radiation Therapy Oncology Group/European Organization for Research and Treatment of Cancer [9], adapted here to the nomenclature proposed by Burnet and colleagues [4] in order to facilitate communication among groups studying normal tissue radiosensitivity. The term 'normal range' refers herein to the range of normal tissue reactions observed in typical radiotherapeutic clinics that treat large numbers of patients without genetic syndromes. All of the skin reactions observed in our study fell within the normal range, and no over-reactors were found.

### Radiosensitivity assay

Initial radiation-induced DNA damage in peripheral blood lymphocytes was measured as described elsewhere [17,18] and was considered an indicator of the molecular radiosensitivity of the normal cells studied. Early and late skin side effects were assessed as mentioned above.

### Early side-effect data

The unit of analysis was a group of 108 patients treated with radiotherapy for curative purposes after breast surgery. The most frequent acute complications found were erythema (91.7%), dry desquamation (29.6%), and moist desquamation (35.2%). According to the score system summarized in Table 1, approximately 13% of patients were classified as highly radiosensitive. Early side effects on the skin might be considered an indicator of clinical radiation sensitivity, and their intensity, score, and distribution have been previously described [9].

### Late side-effect data

**Figure 1:**
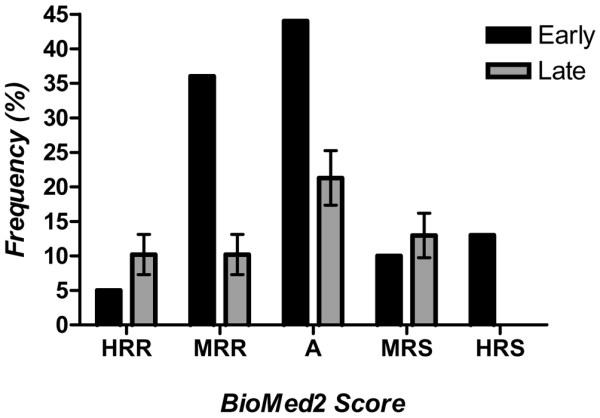
Frequency distribution of skin reactions in women with breast cancer treated postoperatively with radiotherapy. Reactions were classified as early (if observed at the end of the radiotherapy, 108 women) or late (if observed at the 7-year follow-up, 60 women). X-axis Radiation Therapy Oncology Group scoring system modified using the terminology proposed by Burnet (4): A, average; HRR, highly radioresistant; HRS, highly radiosensitive; MRR, moderately radioresistant; MRS, moderately radiosensitive.

Although a significant proportion of the variation in response of normal tissues could be attributed to treatment-related factors, our results showed that dose effects were not sufficient to explain the differences between patients in their skin response (data not shown). Our team previously reported an adequate correlation between scoring of radiation-induced acute skin effects by direct observation and scoring after examination of photographic images, supporting the accuracy of the direct observation of lesions of normal tissue. Therefore, this direct-observation method was used for the assessment of late normal tissue changes in the 60 patients studied, as follows: on the day programmed for the late follow-up, a single physician (EL) generated a report based on direct clinical observation of the whole treated skin, scoring the degree of reaction on the scale used (Table 1, Fig. 1).

### Comparison of *in vitro* and *in vivo* results

A two-sided Student's *t*-test was used to compare mean values of initial radiation-induced DNA damage between the patient groups. Contingency tables and the χ^2 ^test were used to assess any relation between early and late effects.

The relations between *in vivo *and *in vitro *results were studied using a nonparametric regression test, and Spearman's ρ correlation coefficient was calculated. The Statistical Package for Social Sciences (SPSS 11.5) was used for all data processing. Graphics and basic biostatistics were obtained using Graphpad (GraphPad Software Inc, San Diego, CA, USA).

## Results

### Radiosensitivity test

Initial radiation-induced DNA damage was determined in lymphocytes from 108 breast cancer patients after γ-irradiation. The parameter selected was the estimated number of dbs per Gy and per DNA unit [21]. It should be noted that the results obtained from the reference sample of patients included in this paper matched the results obtained in lymphocytes from other breast cancer patients analysed at our laboratory in ongoing studies. [6]. The mean value ± the standard error of the mean was 1.83 ± 0.18 double-strand breaks per Gy.

### Early radiation-induced injury

Assessment of clinical radiation sensitivity was based on the acute skin reactions to the radiotherapy measured [9] on the last day of treatment. Five patients (4.6%) with no adverse side effects were classified as highly radioresistant; 36 (33.3%), 44 (40.7%), and 10 patients (9.3%) with mild to moderate skin reactions were classified as, respectively, moderately radioresistant, average, and moderately radiosensitive; and 13 patients (12%) with pronounced signs of radiation acute sensitivity were considered highly radiosensitive (Fig. 1). The correspondence between the descriptive terms and the radiation sensitivity data is summarized in Table 1. Acute effects on the skin included in the treatment field, such as erythema or desquamation, normally resolve rapidly in most patients. Individual variation in the level of normal tissue response could be theoretically interpreted by the classical sigmoid dose–response curve. Comparison of collateral effects between the surgical treatment subgroups (mastectomy versus breast-conserving therapy) showed that radiation-induced acute toxicity on the skin of the breast cancer patients has the same frequency and intensity regardless of the surgical approach, even when the use of concurrent chemotherapy was taken into consideration [9].

### Overall survival and actuarial probabilities of normal tissue sequelae

Data of survival and late morbidity records were obtained for 87 patients who had undergone radiotherapy treatment for >7 years, of whom 51 were free of cancer disease; 9 were living with disease, 22 had died, and 5 who had received reconstructive surgery were not assessed. A total of 21 patients were missing from the follow-up. Seven years after treatment, the actuarial overall survival of the whole series of breast cancer patients was 48.84 ± 7.62% (mean ± standard error of the mean).

The actuarial probabilities of late radiation side effects, expressed as percentages ± standard errors of the mean, were 10.19 ± 2.91 for highly radioresistant; 10.19 ± 2.91 for moderately radioresistant, 21.30 ± 3.94 for average, 12.96 ± 3.23 for moderately radiosensitive, and 0.0 for highly radiosensitive. Fig. 1 depicts the distribution of the frequency of acute and late effects according to the severity. The distributions of the severity of early and late effects differed. Statistical comparison between early and late collateral effects in the same group of patients gave a χ^2 ^value of 22.38 (*P *= 0.0002), demonstrating a very different distribution frequency between radiation-induced acute toxicity and radiation-related late morbidity.

### Correlation between radiobiological test and early radiation skin side effects

**Figure 2:**
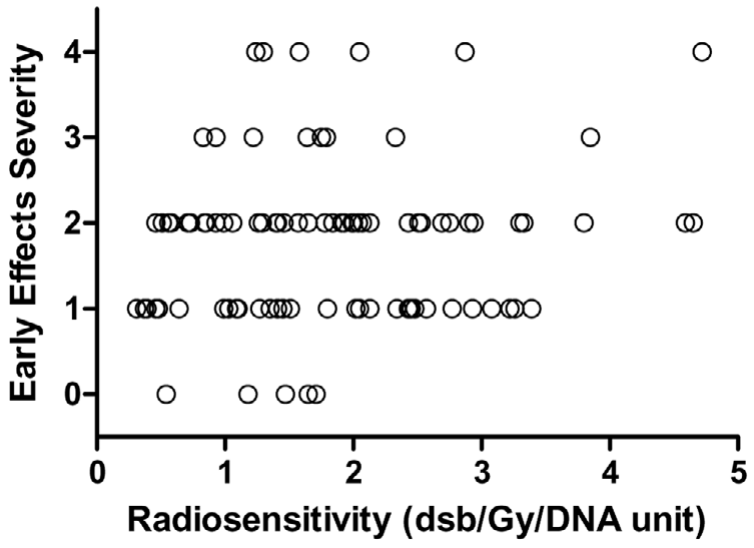
Relation between severity of early radiotherapy-induced skin morbidity and lymphocyte molecular radiosensitivity. Skin morbidity in 108 women was assessed on the treated skin using the scoring system summarized in Table 1. Lymphocyte molecular radiosensitivity was measured as DNA double-strand breaks (dsb) by dose unit (Gy) and DNA unit (200 Mbp). bp, base pairs.

The distribution of early normal tissue reactions observed in this study could be considered approximately normal in shape (Fig. 1). The distribution of the lymphocyte radiosensitivity measured *in vitro *could also be considered approximately Gaussian [6,18]. This similarity prompted us to examine whether the same relation could be found between the number of initial radiation-induced DNA double-strand breaks and the severity of acute adverse skin effects. No relation was found (Fig. 2) between the molecular radiosensitivity values in lymphocytes and the early normal tissue reactions observed *in vivo *(Spearman ρ = 0.076; 95% confidence interval, -0.149 to 0.293; two-tailed *P *= 0.497).

### Correlation between radiobiological test and late radiation skin side effects

**Figure 3:**
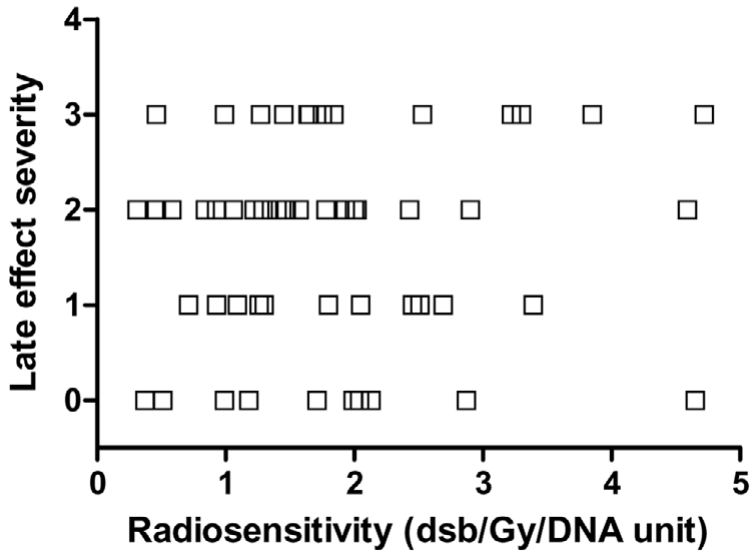
Relation between severity of late radiotherapy-induced skin morbidity and lymphocyte molecular radiosensitivity. Skin morbidity in 60 women was assessed on the treated skin using the scoring system summarized in Table 1. Lymphocyte molecular radiosensitivity was measured as DNA double-strand breaks (dsb) by dose unit (Gy) and DNA unit (200 Mbp). bp, base pairs.

**Figure 4:**
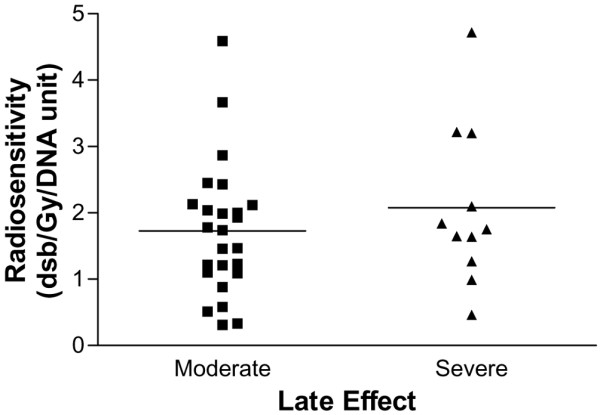
Scatter-plot of quantified late skin reactions of patients and the corresponding *in vitro *radiosensitivity values. Horizontal solid lines are the mean values for each group of patients with breast cancer. Moderate (*n *= 38), women with reactions scored as highly or moderately radioresistant or as having an average response; severe (*n *= 12), patients with reactions scored as highly radiosensitive.

The distribution of late adverse effects observed in these patients does not appear to fit a Gaussian distribution (Fig. 1), and no statistical relation was found between the radiosensitivity test results and the late effects assessed (Fig. 3). No significant relation was found between the *in vitro *assay results and the severity of late side effects (Spearman ρ = 0.063; 95% confidence interval, -0.219 to 0.335; two-tailed *P *= 0.655). Considering the patients with tolerable late effects (highly or moderately radioresistant or with average radioresistance) separately from those with more severe effects (moderately or highly radiosensitive) in a scatter plot, it appears (Fig. 4) that the molecular radiosensitivity assay did not distinguish patients at different levels of risk of developing more severe late skin reactions after radiotherapy treatment.

### Correlation between early and late skin effects

**Figure 5:**
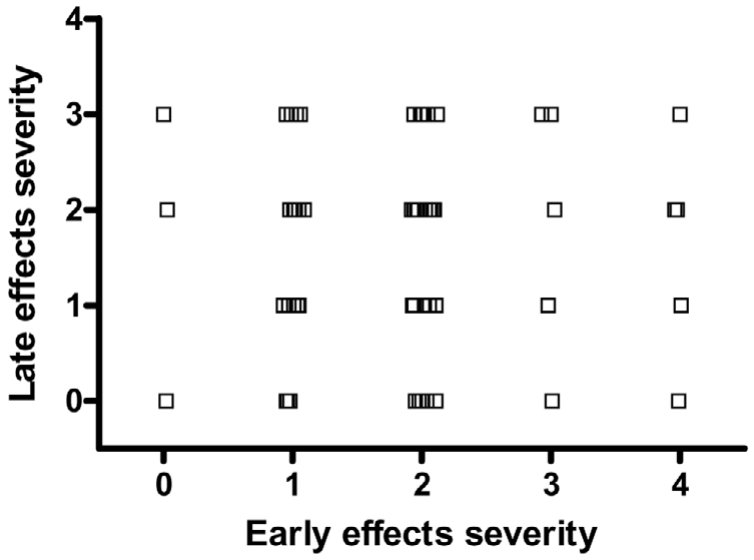
Relation between the severity of early and late side effects of radiotherapy for breast cancer. Women with breast cancer (*n *= 60) were treated postoperatively with radiotherapy and assessed using the scoring system summarized in Table 1.

The data on the severity of early and late adverse effects after radiotherapy for breast cancer showed no relation between these toxic effects (Fig. 5). According to our results, acute and late radiation-related morbidities are independent adverse effects, (Spearman ρ = 0.032; 95% confidence interval, -0.233 to 0.293; two-tailed *P *= 0.809).

### Correlation between early and late effects and treatment volume

**Figure 6:**
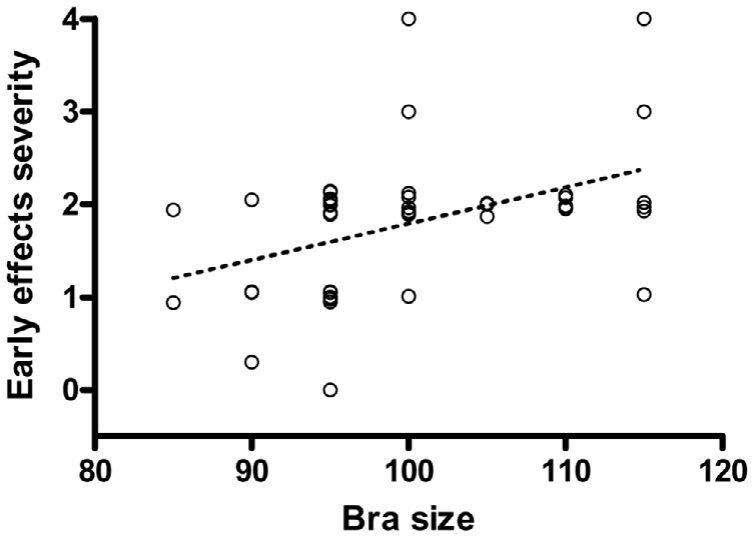
Relation between estimated irradiation volumes and severity of early effects of radiotherapy for breast cancer. Irradiation volumes were estimated from the women's bra size, and the severity of the early effects were scored in the same women (n = 50). Dotted line shows the corresponding regression line (*P *< 0.001).

**Figure 7:**
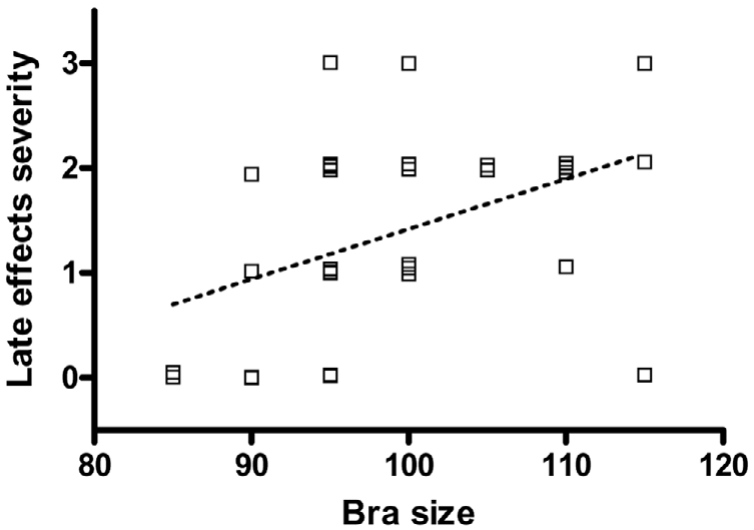
Relation between estimated irradiation volumes and severity of late effects of radiotherapy for breast cancer. Irradiation volumes were estimated from the women's bra size, and the late effects were scored in the same women (n = 33). Dotted line shows the corresponding regression line (*P *= 0.018).

It has classically been reported that patient skin tolerance may be lower with larger breast size. In the present study, this relation was studied in a group of patients treated with breast-conserving surgery, estimating the breast volume from the bra size. When acute adverse effects were considered in 47 patients, a positive relation was found (Spearman ρ = 0.497; 95% confidence interval, 0.236 to 0.691; two-tailed *P *< 0.001) (Fig. 6). However, the relation was weaker when late side effects were considered (Spearman ρ = 0.423; 95% confidence interval, 0.070 to 0.682; two-tailed *P *= 0.018), perhaps because of the smaller number of cases (*n *= 31) analysed (Fig. 7).

## Discussion

In this study, early and late complications in normal tissue were assessed at an arbitrary single point. In this situation, a relative scale of normal tissue reactions, such as the score system proposed by Burnet [4], has a number of advantages over an absolute one. The main objectives of our study were to identify patients with extreme reactions within the normal range and to compare the results of an *in vitro *radiosensitivity test with the severity of acute and late reactions in the same patients. By using this relative scale, we were able to meet these objectives. The concept of the predictive testing of normal tissue reactions in order to individualize radiotherapy prescriptions is founded on a hypothetical relation between the radiosensitivity of cells and that of normal tissue. Although we are inclined to support this hypothesis, the test applied in the present study, based on the initial radiation-induced DNA damage, proved inadequate for use in the individualization of radiotherapy therapy.

Early effects such as erythema and desquamation usually appear during or immediately after radiotherapy therapy, whereas late effects develop some years afterwards. The acute side effects resolve rapidly without treatment [11]. However, in a substantial group of patients, radiation-induced fibrosis, telangiectasia, and skin pigmentation disorders appear at different times after radiotherapy. Generally, the course of radiation sequelae follows a distinct clinical pattern. An erythematous rash can develop on the skin of treated patients within a few hours of exposure and can persist or slowly worsen until the end of radiotherapy treatment. This situation is transient in nature. In severe cases, subepidermal blisters and ulcers may develop. Most of the injuries heal, although the expression of radiation-induced effects can reappear in some individuals after a latency period. Late damage becomes more severe, progresses with time, and usually cannot be halted or reversed [22]. The inability to predict the length of the latency period creates a major problem for the management of these patients. A better understanding of individual variations in normal tissue reactions, which determine tolerance, may allow the individualization of radiotherapeutic prescriptions and improve outcomes. The lag time to the onset of initial late effects might be expected to yield information on the mechanisms underlying the development of late radiation sequelae. Extreme side effects of radiotherapy, including an increased cancer risk after radiation, were observed in patients with inherited disorders such as ataxia-telangiectasia and Nijmegen syndrome [16]. According to the present results, there appears to be no mechanistic relation between the early and late adverse effects of radiation treatment. We speculate that these differences may arise because the healing of acute injuries is a deterministic process whereas late side effects may be stochastic phenomena.

Unconventional, more aggressive irradiation protocols are usually associated with an aggravation of acute reactions that might be related to more severe late effects. Therefore, amelioration of the acute response to radiation has been proposed as a useful approach to minimize late side effects of effective radiation therapy. This proposal assumes a relation between acute and late effects via a non-healing acute response component that directly progresses to a late effect [23]. However, the present results do not support the hypothesis that late effects in normal tissue can be predicted from the acute reactions observed in the same patients.

It also proved impossible in the present study to predict acute or late effects from the results of an *in vitro *assay to measure initial radiation-induced DNA damage. Until recently, it has been generally accepted that the genotoxic consequences of radiation exposure derive from the damage inflicted directly by radiation, producing irreversible changes during DNA replication or cell division or during the processing of DNA damage by enzymatic repair processes [24]. However, there is now considerable evidence that cells that are the progeny of exposed cells but that are not themselves exposed may divide, express delayed gene mutations, and carry chromosomal aberrations. This effect, known as radiation-induced genomic instability, may be expressed via delayed lethal mutations [25], causing prolonged perturbation of tissue volume within the radiation field [26]. Although the mechanisms of those delayed effects of ionizing radiation are unclear, excessive production of reactive oxygen species has been implicated [27]. Recent experiments showed that macrophage activation and neutrophil infiltration are consequences of the recognition and clearance of radiation-induced apoptotic cells and that increased phagocytic cell activity persists after removal of apoptotic bodies. It was demonstrated, contrary to expectations, that the recognition and clearance of apoptotic cells after exposure to radiation produces persistent macrophage activation and a genotype-dependent inflammatory-type response [28]. These phenomena and radiation-induced genetic changes may be important determinants of the longer-term consequences of radiation exposure [28]. Moreover, new evidence suggests that cytokine-mediated multicellular interactions initiate and sustain the fibrogenic process [29,30] that is a long-term effect of radiotherapy.

Initial DNA damage and post-radiation cell survival after radiation have been directly related in *in vitro *experiments [31]. The present findings indicated that the level of radiation-induced DNA damage in normal cells was not a major determinant of the severity of early skin injury. Moreover, no relation was found between the acute injuries and the late sequelae that, after an undetermined latency period, became a burden, lessening the quality of life of these patients [32].

However, a significant correlation has been demonstrated, using new methodologies, between five single-nucleotide polymorphisms (SNPs) and the risk of radiation-induced normal tissue reactions in a small group of breast cancer patients [33]. In fact, the completion of the human genome project and the availability of novel and powerful technologies in genomics, proteomics, and functional genomics promise to have a major impact on clinical practice. These developments are likely to change the way in which diseases will be diagnosed, treated, and monitored in the near future. Cancer, as a complex disease that affects a significant proportion of world population, has become a prime target of novel technologies, often referred to as 'omic' platforms, and it is anticipated that progress will be made towards a predictive, individualized approach to cancer care. One area of knowledge where advances are expected is on the complex variability in normal tissue radiation response, which depends on the interaction of multiple gene products. There is a growing shift from the study of single parameters of molecular or cellular radiosensitivity to the analysis of complex biological systems, and one of the main challenges we face is how best to apply the 'omic' technologies to clinically relevant samples in a well-defined clinical and pathological framework. An example of this type of venture is the European GENEPI project [34], which aims to study a large cohort of patients under highly controlled and standardized radiotherapy conditions.

## Conclusion

Our first conclusion is an experimental one. These results do not support the hypothesis that the response of normal tissue to radiation can be predicted by an *in vitro *test. This conclusion was reached by other authors [8,10], although some results in defence of this hypothesis have also been published [35,36]. A possible explanation is that *in vitro *cellular radiosensitivity tests and molecular DNA damage assays do not take account of the variable degree of cytokine response, tissue remodeling, and collagen deposition that may characterize the specific normal-tissue response of each patient [29]. The paradigm that radiotherapy effects are restricted to the direct or indirect effects of radiation-induced DNA damage is challenged by the present results, which indicate that early and late effects can also be induced by unexpected interactions between irradiated and nonirradiated cells (bystander effects). This conclusion is supported by published results that showed a clear relation between the severity of late toxicity in radiotherapy treatment and the volume of normal tissue included in the field of treatment, although a significant correlation was found between breast size and dose inhomogeneities that may account for the marked changes in breast appearance reported in women with large breasts [37].

Our second conclusion is a theoretical one, and takes the form of a proposal to change the model adopted in radiobiological studies to date. Thus, for teaching and research purposes, 'direct action' could be defined as all physicochemical processes that occur after energy cession from the ionizing radiation to the tissues. Within this concept would be included actions produced by free radicals that result from the interaction of radiation with the water molecules – that is, the effects hitherto designated indirect radiation action on the DNA molecule. The cellular consequences of the direct action of radiation in terms of lethal and potentially lethal damage to DNA can be explained by linear-quadratic radiation cell survival models. However, these models cannot explain the late adverse effects of radiation, and a more general theory appears to be required.

A few days after the end of radiation treatment, cells within the irradiated volume can act in one of three ways: they can grow and divide, the basis for the healing of acute injuries; they can not grow but stay alive; or they may survive for a long time with important immunological changes, disappearing by apoptosis or apoptosis-like cell death very slowly and becoming a chronic focus of immunological system stimulation that could produce the late actions observed. Therefore, indirect action could be considered the whole immunological response of the body to the stress induced by radiation in the target volume. This may produce late side effects of varying severity that in a stochastic fashion, through a time-dependent probability relation, could lead to a lifelong risk of developing late complications [14,32,38]. In this relation, the volume of tissue irradiated may be a multiplicity constant of the frequency and severity of the late side effects. Patients and clinicians should be aware of these aspects of radiotherapy therapy. The study of these immunological changes is complex but could, given the human genome data now available, offer a key to improving radiotherapy outcomes in cancer patients.

Finally, our group supports the view that the risks of radiotherapy can be fully understood only after long-term follow-up studies. An important research aim is to develop a test that can predict late side effects.

## Competing interests

The author(s) declare that they have no competing interests.

## Authors' contributions

EL was significantly involved in patient recruitment, patient treatment, and assessment of late normal tissue responses; contributed to the correlation of clinical data with experimental findings; and took a role in supervising the final version of article. RG and RdM were significantly involved in patient recruitment and patient treatment and in the assessment of skin reactions. JMG participated in patient treatment. MIN, MV, and MTV carried out the *in vitro *radiosensitivity test and took a role in the discussion of results. JAMG, DMO, and FJO carried out part of the laboratory work. JMRdA conceived and designed the study, interpreted the data, and revised the paper, giving final approval of the version to be submitted. All the authors read and approved the final manuscript.

## Figures and Tables

**Table 1:** Scoring system used to document cutaneous and subcutaneous reactions in breast cancer patients receiving radiotherapy

Grade	Early reactions^a^	Late reactions^b^
Grade 0 – Highly radioresistant patients	No toxicity observed: no erythema, desquamation, or pain	Absence of differences between irradiated and nonirradiated skin
Grade 1 – Moderately radioresistant patients	Faint, dull, or bright erythema, psilosis, dry desquamation, mild oedema	Minimal telangiectasia, slight breast asymmetry, mild hyperpigmentation
Grade 2 – Patients with average radiosensitivity	Severe erythema, at least one moist desquamation of small size, moderate oedema	Marked telangiectasia, moderate hyperpigmentation, increased density and palpable firmness, mild oedema
Grade 3 – Moderately radiosensitive patients	Severe or confluent moist desquamation	Partially confluent telangiectasia, severe hyperpigmentation, severe oedema, subcutaneous fibrosis with fixation
Grade 4 – Highly radiosensitive patients	Ulceration, haemorrhage	Totally confluent telangiectasia, very marked density, retraction and fixation. Major aesthetic sequelae in treated breast

^a^Morbidity assessed at the end of radiotherapy treatment. ^b^Morbidity assessed at end of 7-year follow-up period
